# Evaluation of a smartphone electrocardiograph in healthy foals and comparison to standard base-apex electrocardiography

**DOI:** 10.1007/s11259-023-10206-x

**Published:** 2023-11-10

**Authors:** Francesca Bindi, Tommaso Vezzosi, Giulia Sala, Francesca Freccero, Paola Marmorini, Francesca Bonelli, Micaela Sgorbini

**Affiliations:** 1 Department of Veterinary Sciences, University of Pisa, via Livornese, San Piero a Grado, 56122 Italy; 2 Department of Veterinary Medical Sciences, Università degli Studi di Bologna, Bologna, 40064 Italy; 3 Private Practitioner, Pisa, Italy

**Keywords:** Accuracy, Cardiology, Electrocardiography, Feasibility, Foal, Smartphone-based ECG

## Abstract

Smartphone-based technology for ECG recording has recently spread as a complementary tool for electrocardiographic screening and monitoring in adult horses and in other animal species. The present study aimed to assess the feasibility and accuracy of a smartphone-based ECG in healthy foals. This was a prospective observational study (authorization n. 45,865/2016) including 22 foals aged less than 21 days. A reference standard base-apex ECG (rECG) was acquired, and a smartphone ECG (sECG) was recorded immediately after by using a smartphone-based single lead electrocardiograph. All ECG tracings were evaluated in a blind fashion by a single board-certified cardiologist, who judged whether the tracings were acceptable for interpretation and performed ECG measurements and diagnosis. The Spearman correlation coefficient, the Cohen’s k test and the Bland-Altman test were used to assess the agreement between sECG and rECG. All sECG tracings were acceptable for interpretation. All foals showed sinus rhythm on both rRCG and sECG tracings, with perfect agreement in heart rate classification (κ = 0.87; p < 0.001). No clinically relevant differences were found in the assessment of waves and intervals duration. Concerning P wave and QRS complex polarity, the percentage of agreement between rECG and sECG was 78% and 83%, respectively. About ECG tracing quality, rECG and sECG showed a substantial agreement (κ = 0.624; p < 0.001). In conclusion, the smartphone-based ECG device tested in the present study recorded good quality single-lead ECG tracings in foals, reliable for heart rate and ECG measurements, but different polarity of P waves and QRS complexes was found in some foals in comparison to rECG.

## Introduction

In foals, a standard ECG is usually performed at rest using the base-apex lead placement. The procedure requires an electrocardiograph with ECG leads, clip or adhesive electrodes, alcohol and/or ECG conductive gel. Recordings can be made with the foal in standing or in lateral recumbency to minimize body movements (Nógrádi 2017).

Wireless, smartphone-based technology for ECG recording has recently spread as a complementary tool for electrocardiographic screening and monitoring in horses (Vezzosi et al. 2018; Kraus et al. 2019; Alberti et al. 2020; Welch-Huston et al. 2020; Vitale et al. 2021), sheep (King et al. 2023), dairy cows (Bonelli et al. 2019), goats (Smith 2020), dogs and cats (Vezzosi et al. 2016; Kraus et al. 2016). In human medicine, smartphone-based one-lead ECG devices have been developed using specific adaptors and software (Saxon et al. 2013; Ho et al. 2014; Haberman et al. 2015; Nguyen et al. 2015; Tarakji et al. 2015).

The present study aimed to assess the feasibility and accuracy of a smartphone-based ECG in healthy foals to evaluate HR and ECG measurements compared with standard ECG.

## Materials and methods

A total of 22 healthy foals were enrolled in this prospective observational study performed during a two-year period (2021–2022). The research protocol was approved by the Institutional Animal Care and Use Committee of the University of Pisa (nr. 45,865/2016). Inclusion criteria were: pregnancy length between 320 and 360 days; unassisted delivery; righting and suckling reflex, sternal recumbency, quadrupedal position, and nursing the mare within reference ranges (Sgorbini 2007). All the foals were considered healthy based on history and physical examination.

### ECG acquisition and analysis

ECGs were recorded only once in foals aged less than 21 days. Foals were conscious, non-sedated, manually restrained in a standing position.

**Fig. 1:**
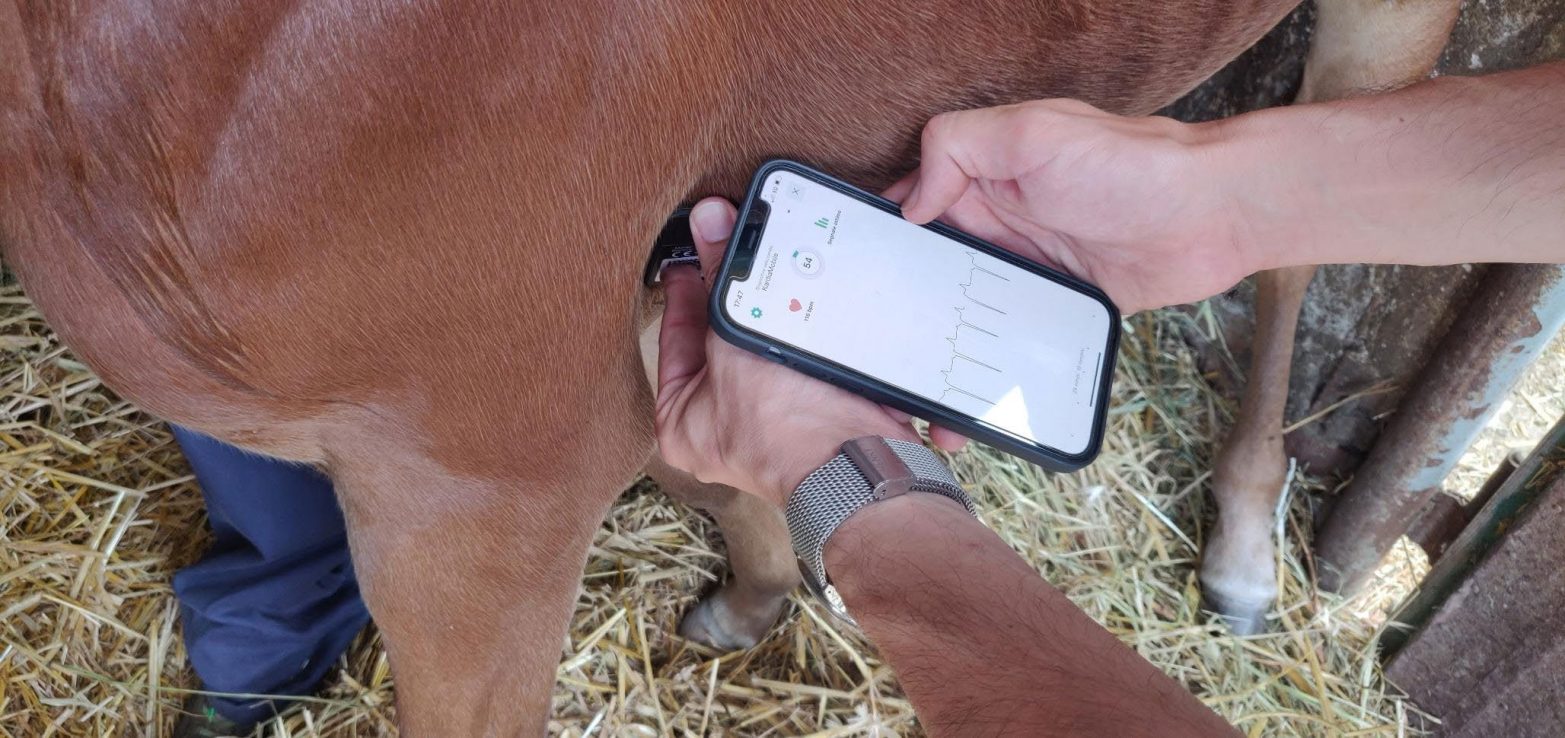
The smartphone-based device was placed on the left chest wall, in the precordial area, slightly below the olecranon, with a dorso-ventral orientation of the device

A reference standard base-apex ECG (rECG) (van Loon 2010) was acquired by a digital telemetric ECG device (Televet 100, Engel Engineering GmbH, Heusenstamm, Germany) for 30 s. Smartphone ECGs (sECG) were recorded immediately after the rECG by using a smartphone-based single lead electrocardiograph (AliveCor KardiaMobile EKG Monitor, AliveCor Inc., USA) with its smartphone application (Kardia, AliveCor Inc., USA). The sECG tracings were recorded using an iPhone 12 (Apple, Cupertino, California, USA). The smartphone-based device was placed on the left chest wall, in the precordial area, slightly below the olecranon, with a dorso-ventral orientation of the device (Fig. 1). As for rECG recording, just a small amount of alcohol was rubbed on the left precordial area to improve the ECG signal quality and no clipping was needed. Smartphone ECG recordings were automatically digitized by the device, sent via email, and stored as a PDF. The same operator recorded both the rECG and the sECG tracing.

All the sECG tracings were masked for subject identity and evaluated in a blind fashion by an experienced veterinary cardiologist (T.V.), who judged whether the tracings were acceptable for interpretation and performed ECG measurements and diagnosis.

All ECGs were quality scored on the basis of the presence or absence of baseline undulation and tremor artifacts using a 3-point scoring system previously described (King et al. 2023). Briefly, score 0 = high-quality recording with no baseline wander or small baseline deflections; score 1 = Intermittent, mild tremors or baseline deflections or mild baseline wander; score 2 = Moderate tremors or baseline deflection consistent throughout the recording; score 3 = severe tremor artifact inhibiting the interpretation of the P and T waves.

The ECG measurements have been performed using lead I on the rECG tracings and the only available lead of the sECG. The mean HR calculated automatically by the smartphone application (App HR) was also recorded. The P wave and QRS complex polarity were evaluated.

### Statistical analysis

Data were assessed for normality using the D’Agostino & Pearson test and the results were expressed as mean and standard deviation or median and range values.

Quality score was analyzed using Spearman test to verify correlation and Cohen’s k test to assess the agreement between sECG and rECG.

Cohen’s κ test was also used to calculate the agreement between the sECG and rECG for HR classification using HR reference interval, as following: normal range for HR was defined as between 80 and 100 bpm, bradycardia was defined when the HR was below 80 bpm, and tachycardia when the HR was greater than 100 bpm (Desrochers 2011). Cohen’s k test was also applied to verify agreement between sECG and rECG for P and QRS polarity. The κ coefficient was interpreted as follows: values ≤ 0.00 as no agreement, 0.00–0.21 as slight, 0.21–0.40 as fair, 0.41–0.60 as moderate, 0.61–0.80 as substantial, 0.81–1.00 as almost perfect agreement. If the contingency table reported one or more values equal to zero, Cohen’s kappa could not be calculated, and thus, in these cases, the percentage of agreement was used.

Using the Bland-Altman test, bias and 95% limits of agreement were calculated for the duration of the P wave, PR interval, QRS complex, and QT interval to verify the differences between the sECG and rECG.

Statistical analyses were performed with commercial software (Microsoft Excel, 2011; GraphPad Prism 6, USA). A P value of < 0.05 was considered significant.

## Results

### Animals, feasibility and accuracy

The study included 22 trotter foals with a median age of 8 days (range: 1–21 days). Thirteen out of 22 foals were fillies (59.1%) and 9/22 (40.9%) were colts. The handling was well tolerated in all the foals included.

Among sECG tracings, 4/22 (18%) showed score 3 and, thus were judged non-acceptable for interpretation and excluded from the study analysis. Overall, a total of 18/22 ECG tracings (82%) were included in the analysis.

The rECG traces was scored 0, 1 and 2 in 4/18 (22%), 6/18 (33%), and 8/18 (45%) cases, respectively. The sECG recording quality scored 1 in 8/18 (45%) and 2 in 10/18 (55%) traces. The Spearman correlation coefficient was 0.836 (p < 0.001) between rECG and sECG tracing quality and the weighted k was 0.624 showing a substantial agreement (95% CI: 0.422–0.825; p < 0.001).

**Fig. 2:**
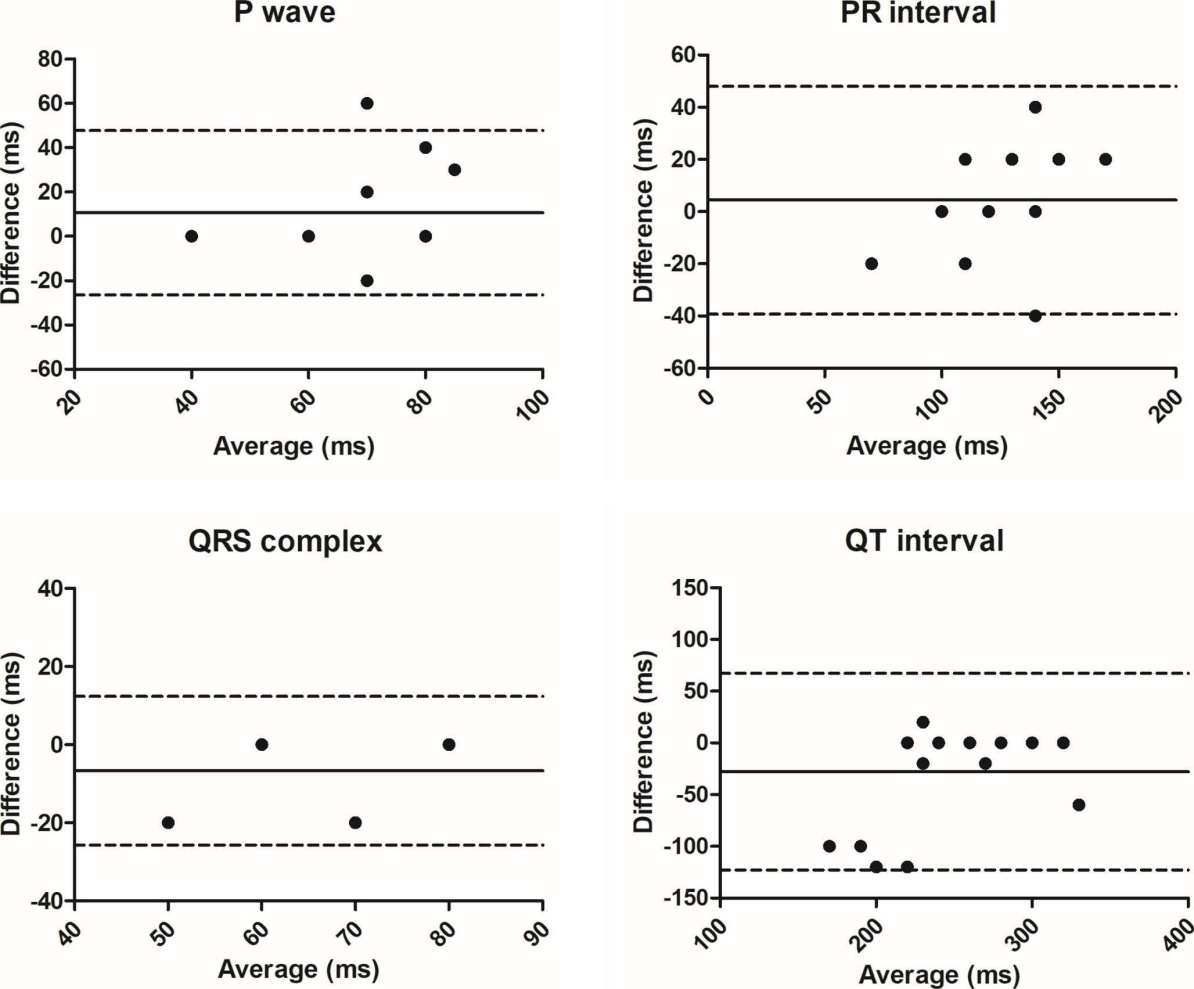
Bland-Altman plots of the difference in electrocardiographic measurements (P wave, PR interval, QRS complex and QT interval duration) between standard ECG and smartphone ECG.

Regarding heart rhythm, on both rRCG and sECG tracings, all the foals showed sinus rhythm. Results on waves and intervals duration are reported in Table 1; Fig. 2.

**Table 1:** Concordance between reference standard ECG and smartphone ECG in the assessment of waves and intervals duration

	Bias	95% LOA
P (ms)	10.6	-26.5; 47.7
PR (ms)	4.4	-39.2; 48.1
QRS (ms)	-6.7	-25.7; 12.4
QT (ms)	-27.8	-123.0; 67.4

Data are reported as bias and 95% limits of agreement (LOA).

The HR was assessed in all the foals on rECG and on sECG when manually measured on digitized tracing, while HR was registered by the App HR in 16/18 (88.9%) foals. According to the rECG, 5/18 foals (28%) had a normal HR, 13/18 (72%) had tachycardia, and no foals showed bradycardia. According to sECG, tachycardia was present in 12/18 foals (67%), while 6/18 (33%) animals showed normal HR. An almost perfect agreement (κ = 0.87; 95% CI: 0.623–1.116; p < 0.001) between the rECG and the sECG was found in the HR classification when it was manually measured on digitized tracings. According to HR registered by the app, normal HR was found in 6/16 foals (37.5%), while tachycardia was diagnosed in 10/16 (62.5%), leading to a substantial agreement between HR measured on rECG tracings and the App HR (κ = 0.74; 95% CI: 0.359–1.069; p = 0.003).

**Fig. 3:**
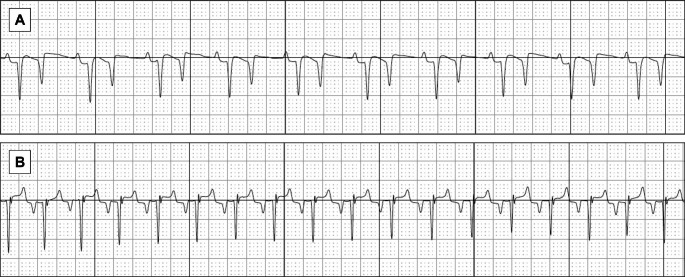
Smartphone ECG tracings showing different P wave polarity in two foals of the study; positive polarity in (**A**) and negative polarity in (**B**). Paper speed = 25 mm/s and 20 mm/mV

On the rECG tracings, the P polarity was always positive (100%), while on the sECG tracings, the P polarity was positive in 13/18 (72%) and negative in 5/18 (28%) cases (Fig. 3). The percentage of agreement was 77.8%.

The QRS complexes showed a negative polarity on rECG in all cases (100%); differently, on the sECG tracings, the QRS polarity was negative in 15/18 cases (83%) and positive in 3/18 (17%). The percentage of agreement was 83.3%.

## Discussion

The present study aimed to assess the feasibility and accuracy of a smartphone-based ECG in foals, and its accuracy to evaluate HR, heart rhythm and ECG measurements compared with standard ECG. To the best of the authors’ knowledge, no studies on the use of smartphone ECG in foals have been previously performed. Overall, (1) sECG was feasible in foals manually restrained, (2) most sECG tracings were judged interpretable, and (3) a substantial to high accuracy was found for HR, waves, and intervals in sECG tracing.

In our investigation, the sECG tracings were interpretable in 82% of cases, a lower value in comparison to humans (87-99.6%) (Saxon et al. 2013; Tarakji et al. 2015), dogs (89%) (Vezzosi et al. 2019), dairy cows (89%) (Bonelli et al. 2019), and adult horses (91–96%) (Vezzosi et al. 2018; Alberti et al. 2020). The differences may be related to a certain restlessness of foals when handled as compared to adult animals. In a study assessing feasibility of smartphone-based ECG recording in sheep, a lower percentage of interpretability was found (65%) (King et al. 2023), reasonably because of the feral temperament of the animals enrolled in the study.

Moreover, consistent with studies in small ruminants (Smith 2020; King et al. 2023), the quality of sECG traces was found to be lower than those of rECG traces. This finding may be attributed to inherent difficulty in maintaining optimal contact of electrodes with skin when using the smartphone-based device compared to the digital telemetric ECG device. However, the device allowed identification of normal sinus rhythm in all foals, and, in contrast to findings from previous studies in sheep and goats (Smith 2020; King et al. 2023), our results show a substantial agreement between rECG and sECG tracing quality.

In our study, a substantial agreement was found between the App HR and the rECG (k = 0.74). However, in many sECG tracings, the App HR was overestimated. This is widely reported in previous studies in which the reliability of smartphone electrocardiography was also assessed (Vezzosi et al. 2016, 2018; Kraus et al. 2019; Bonelli et al. 2019; Alberti et al. 2020). The overestimating values of App HR could be related to an “oversensing” of the artifacts or an erroneous identification of P and T waves as R waves (Kraus et al. 2019).

A percentage of agreement of around 80% was found for both P wave and QRS complex polarity. This finding was in accordance with what reported in goat (Smith et al. 2020), but not in line with what found in cattle (Bonelli et al. 2019) and in adult horses (Vezzosi et al. 2018; Alberti et al. 2020). In those previous studies, a good agreement was found between rECG and sECG for QRS polarity, but not for P polarity. A potential reason for the observed discrepancy was that the single-lead of the smartphone-based device is basically a precordial lead that assesses a different anatomic plane in comparison in lead I of the rECG, possibly leading to P waves of different amplitude and polarity.

The present study has some limitations. Firstly, the ECGs were not recorded simultaneously due to the difficulty in adequately performing both recordings. This impacted the assessment of reliability of the smartphone device in measuring HR and the duration of ECG waves and intervals. Second, the same operator recorded both the rECG and the sECG while another blinded operator assessed the suitability for interpretation. Therefore, the inter-operator variability in the quality of ECG recording and interpretation was not evaluated. Third, the study group was small. Lastly, no arrhythmias were found in the present study sample, so no statistical analysis could be performed to evaluate the smartphone ECG’s diagnostic efficacy for finding cardiac arrhythmias.

## Conclusions

In conclusion, the smartphone-based ECG device tested in the present study recorded good quality single-lead ECG tracings in healthy foals, reliable for measuring HR and wave and intervals durations, but different polarity of P waves and QRS complexes was found in some foals in comparison to standard ECG. Smartphone ECG devices could represent a reliable diagnostic tool for electrocardiographic screening in foals, especially under field conditions.

## Data Availability

The datasets in this study are available from the corresponding author on reasonable request.
